# Content Analysis of Stroke Teleconsultation Recordings in the Moravian-Silesian Region, Czech Republic

**DOI:** 10.3389/fneur.2021.664918

**Published:** 2021-09-09

**Authors:** Linda Kasickova, Katie Lin, Ondrej Volny, Martin Cabal, David Holes, Michael D. Hill, Michal Bar, Robert Mikulik

**Affiliations:** ^1^Department of Neurology, University Hospital Ostrava, Ostrava, Czechia; ^2^Faculty of Medicine, Masaryk University, Brno, Czechia; ^3^Departments of Clinical Neurosciences, Calgary Stroke Program, Cumming School of Medicine and Hotchkiss Brain Institute, University of Calgary, Calgary, AB, Canada; ^4^Department of Community Health Sciences, Cumming School of Medicine, University of Calgary, Calgary, AB, Canada; ^5^Faculty of Medicine, Ostrava University, Ostrava, Czechia; ^6^International Clinical Research Centre, Stroke Research Program, St. Anne's University Hospital, Brno, Czechia; ^7^Czech National Centre for Evidence-Based Healthcare and Knowledge Translation (Cochrane Czechia, Czech EBHC: JBI Centre of Excellence, Masaryk University GRADE Centre), Institute of Biostatistics and Analyses, Faculty of Medicine, Masaryk University, Brno, Czechia; ^8^First Faculty of Medicine, Charles University in Prague, Prague, Czechia; ^9^Emergency Medical Service of Moravian-Silesian Region, Ostrava, Czechia; ^10^Jessenius Faculty of Medicine in Martin, Commenius University in Bratislava, Bratislava, Slovakia; ^11^Department of Neurology, Faculty of Medicine, St. Anne's University Hospital, Masaryk University, Brno, Czechia

**Keywords:** ischemic stroke, prehospital care, emergency medical service, prenotification, teleconsultation

## Abstract

**Background:** Direct teleconsultations between emergency medical services (EMS) crews and hospital-based stroke neurologists are mandated in the Czech Republic as triage and prenotification tool in acute stroke patients. The main aim of this study was to analyze the efficacy as well as quality of such teleconsultations in daily clinical practice.

**Methods:** This is a descriptive analysis of teleconsultations between EMS paramedic crews and hospital-based neurologists in a geographically defined region of the Czech Republic (Moravian-Silesian region) between October 2018 to December 2018. All teleconsultations were analyzed for length and content. Content analysis included the following information: date, age, sex, prehospital neurological deficit(s), known/unknown time of symptom onset, anticoagulation status, vital signs, premorbid disability, and patient ID/insurance company number.

**Results:** Within the study period, paramedics conducted 522 calls across 6 stroke centers. Of these, 334 (64%) calls were conducted because patients met pre-established prehospital criteria for suspected acute stroke. Median call duration was 1 min 44 s ± 56 s (minimum 50 s, maximum 5 min 5 s). Amongst the analyzed prehospital teleconsultations, stroke onset time was reported in 95% of cases, neurological deficit in 96%, significant co-morbidities in 53%, premorbid disability in 37%, and anticoagulation status in 53%.

**Conclusion:** Teleconsultations between paramedics and hospital-based neurologists are not time-consuming. Stroke onset time and severity of neurological deficit are consistently communicated, however other important information such as comorbidities, premorbid disability, and anticoagulation status are reported inconsistently.

## Introduction

Acute ischemic stroke is a medical emergency with effective but time-limited treatment including intravenous thrombolysis and/or endovascular (mechanical) thrombectomy. The sooner therapy is provided, the better clinical outcome (1, 2). Every minute of delay in treatment initiation results in an average of 1.8 days of healthy life lost (3).

Prenotification by EMS has been associated with decreased prehospital (4–6) as well as in-hospital times (4, 5, 7–10) and increased thrombolytic administration rates (5, 9, 10). Prenotification by EMS can further facilitate early activation of stroke interventional teams.

Teleconsultation could serve as both a prehospital triage tool (11) and a prenotification (12). Advantages of teleconsultation include the provision of expert guidance for paramedic teams in the prehospital environment, more accurate decision-making for patient transportation decisions, and early activation of ED and stroke interventional teams to reduce treatment delays upon arrival at destination. Proposed disadvantages of teleconsultation include the potential time burden of teleconsultation calls and the inconsistent quality of communicated information. The main goal of this study is to assess the efficacy and measure teleconsultation quality in the management of acute stroke patients.

## Materials and Methods

This is a descriptive observational study of all available teleconsultation events for suspected acute ischemic stroke cases involving prehospital teleconsultations between EMS and stroke neurologists within the geographically defined Moravian-Silesian region [1 comprehensive stroke center (CSC), 5 primary stroke centers (PSC), catchment area: 1.2 million inhabitants] between October 2018 to December 2018. This study assessed audio-recordings of all recorded prehospital communications between the EMS and hospital-based neurologists. The Ethics Committee of the University Hospital Ostrava approved the study.

### Organization of Stroke Services in the Czech Republic

There are currently 13 comprehensive stroke centers (CSC) performing endovascular therapy and 32 primary centers capable of administering intravenous thrombolysis (IVT) in the Czech Republic.

Based on legislation in the Czech Republic, every suspected stroke case must be tele-consulted with a hospital-based neurologist. EMS providers are trained to activate stroke protocols if a patient meets the following criteria: sudden onset of neurological deficit (1 major symptom—hemiparesis/plegia, facial droop or speech disturbances or 2 following minor symptoms—hemihypesthesia, dysarthria, hemianopsia, loss of consciousness, diplopia, atypical “worst-ever” headache, meningism, or vertigo with nausea and vomiting) with sudden onset and last seen normal in the past 24 h. EMS providers are trained regularly by stroke physician (i.e., PowerPoint presentation/webinar with testing of knowledge at the end of session. Ideally, EMS personnel should convey all relevant information to primary treating physician (i.e., stroke neurologist) in order to make patient-centered decisions about transport and treatment strategy (13). Teleconsultation represents a critical opportunity to provide expert-guided, individualized care to every stroke patient.

**Figure 1:**
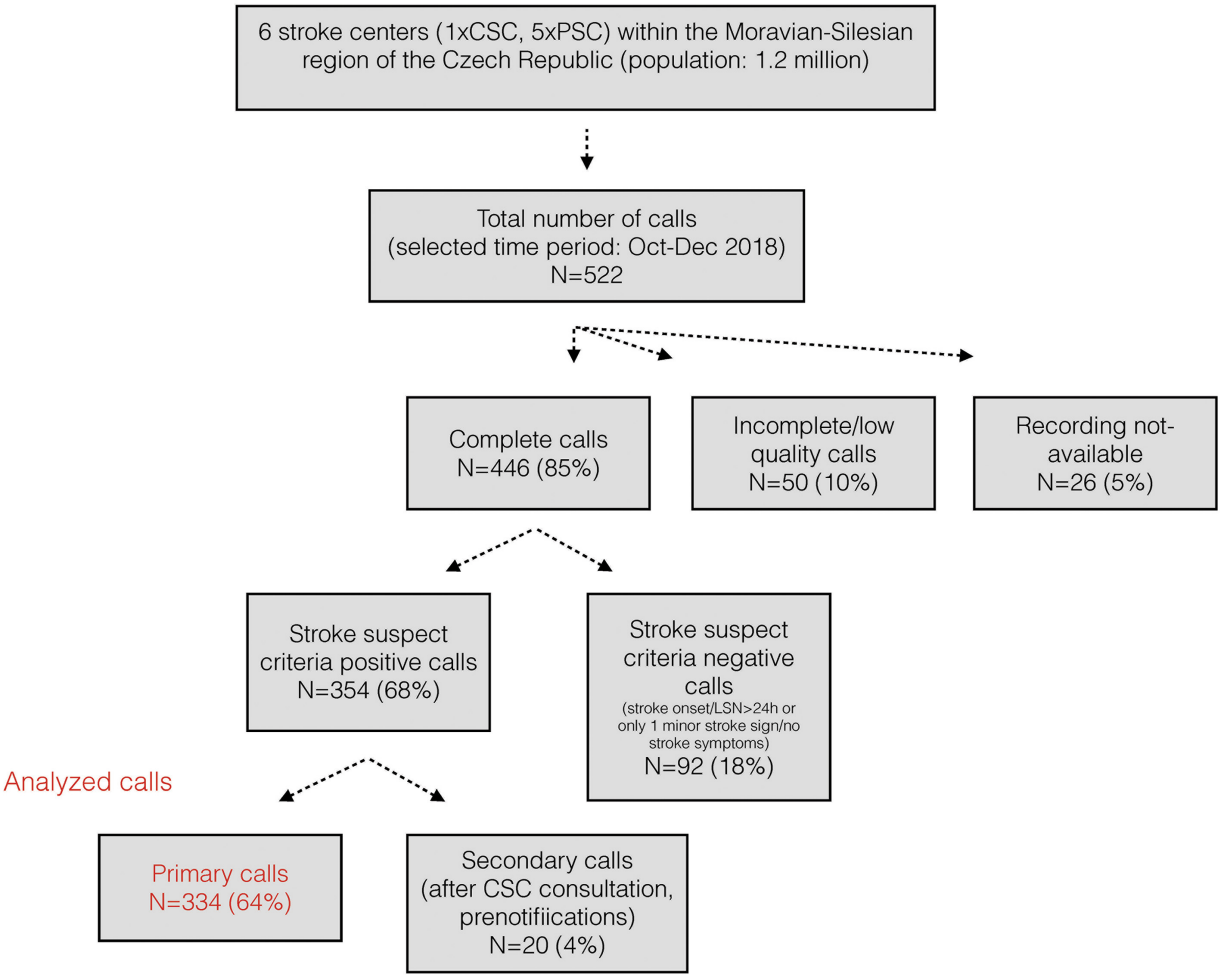
Flow chart of stroke teleconsultation content analysis.

Each of the 14 regions within the Czech Republic has one EMS headquarter. In 2016, the validated prehospital stroke scale called FAST PLUS was implemented in the Moravian-Silesian region to test for potential large vessel occlusion strokes (14). The FAST PLUS test positivity helps to guide the EMS crew and to initiate a teleconsultation with the hospital-based neurologist at the designated comprehensive stroke center (CSC). The stroke team then determines whether the patient is to be transported directly to a CSC or is first to be directed to a PSC. If patient is directed to PSC, EMS prenotification call (i.e., secondary call—for details see the Figure 1) is provided in advance of patient arrival to the receiving stroke team at PSC. Patients with a negative FAST PLUS are teleconsulted with the nearest PSC and this teleconsultation serves also as prenotification. For this study, all available data from the Moravian-Silesian EMS teleconsultations were analyzed.

### Teleconsultations Between EMS and Hospital-Based Neurologist

All teleconsultations between EMS and a hospital-based neurologist are connected via EMS dispatchers and recorded. For the purpose of this study, recorded teleconsultations between EMS crews and hospital-based neurologists from all stroke centers in the Moravian-Silesian region were stored on an encrypted compact disc (CD). Personal data was handled in accordance with Article XIII of the GDPR Regulation.

### Analysis of Teleconsultations

Content analysis was performed by a trained neurologist (L.K). Incomplete (interrupted calls)/low quality calls (calls with technical problems), calls which did not fulfill the criteria of suspected acute stroke (i.e., stroke onset/last seen normal > 24 h or no major stroke sign or only 1 minor stroke sign) and secondary calls (subsequent PSC prenotifications) were excluded (Figure 1). Date and length of calls were recorded. The presence or absence of the following information was collected: age, sex, neurological deficit (1 major symptom—hemiparesis/plegia, facial droop or speech disturbances or 2 minor symptoms—hemihypesthesia, dysarthria, hemianopsia, loss of consciousness, diplopia, atypical “worst-ever” headache, meningism or vertigo with nausea and vomiting), FAST PLUS test positivity (if severe unilateral hemiparesis/hemiplegia is present), stroke onset time/last seen normal/wake-up stroke or unknown stroke onset, pre-morbid status (independent, dependent or modified Rankin Scale, if available), anticoagulation therapy (warfarin or new oral anticoagulation), significant co-morbidities (e.g., prior stroke, history of epilepsy, severe trauma/surgery within last 2 weeks, gastrointestinal bleeding within 3 weeks, cancer), all other co-morbidities (if available), insurance identification number, vital functions measured by paramedics (including blood pressure, level of glycemia, level of consciousness, heart rate, oxygen saturation, heart rhythm). Final diagnosis and treatment of patients transported to the CSC was also collected. Standard descriptive statistics were used to measure the central tendency and variability of baseline characteristics.

## Results

Within study period, there were 889 hospital admissions in the Moravian-Silesian region with diagnosis of any (acute and non-acute) ischemic stroke or TIA. Altogether 522 teleconsultations were recorded during the study period. Of these, 334 (64%) calls were triggered by correct identification of patients meeting pre-established prehospital stroke triage criteria (i.e., stroke onset/last seen normal <24 h or no major stroke sign or only 1 minor stroke sign (please see Figure 1).

**Figure 2:**
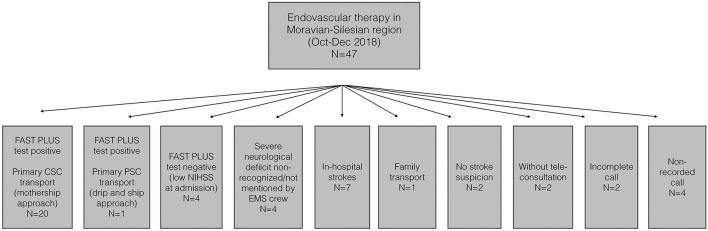
Prehospital routes of EVT patients in the Moravian-Silesian region (October–December 2018).

Altogether 152 (17%) were treated with IVT and 47 (5%) patients underwent EVT. Figure 2 summarizes prehospital routes of EVT patients within study period.

Of 87 patients who were transported directly to a CSC, hospital discharge diagnosis was ischemic stroke in 76%, hemorrhagic stroke in 14%, and stroke mimic in 10%. Twenty-five patients with acute ischemic stroke (AIS) were treated with IVT, another 18 with both EVT and IVT, 2 patients underwent only EVT and 21 patients were treated conservatively.

Median call duration was 1 min 44 s ± 56 s (minimum 50 s, maximum 5 min 5 s). Six percent of calls lasted <1 min and 86% <3 min. Seventy-three percent of calls were conducted during weekdays and 67% during working hours (7:00–17:00).

Stroke onset time was reported in 95% of cases, neurological deficit in 96%, significant co-morbidities in 53%, premorbid disability affecting patient activities of daily living in 37%, and active anticoagulation therapy in 53%. Blood pressure was reported in 48%, level of glycemia in 27%, oxygen saturation level in 8%, heart rate in 8%, level of consciousness in 7%, and heart rhythm in 3% (Table 1).

**Table 1:** Teleconsultation content analysis.

		**Primary calls**
Total No. of calls		334
Duration of call, median (IQR)		01:44 (01:20–02:30)
***Timing of call***	
Weekday	Working hours	76%
	After working hours (>17:00)	24%
Weekend/holidays		27%
***Identification of the patients***	
Reported vs. non-reported		46 and 54%
Identification reported	ID (full name and/or patient identification number)	46%
	Sex, male (%)	49%
	Age, yes/no	100%
*Patients factors*	Initial major and non-major deficit	96 and 4%
	Percentage of FAST PLUS positive patients	19%
	Stroke onset/last seen normal/wake-up stroke symptoms/unknown, yes/no	95%
	Comorbidities mentioned, yes/no	53%
	Premorbid status mentioned, yes/no	37%
	Anticoagulation therapy, yes/no	53%
	Insurance company, yes/no	4%
*Vital functions*	Blood pressure	48%
	Glycemia	27%
	Level of consciousness	7%
	Heart rate	8%
	Oxygen saturation level	8%
	Heart rhythm	3%

## Discussion

Our study analyzed teleconsultations between EMS crews and hospital-based neurologists for all suspected stroke cases. One major finding of our study is that teleconsultation itself does not contribute substantially to any pre-hospital delay in stroke management. The duration of the majority (59%) of calls was between 1 and 2 min, and the most critical elements (stroke symptom onset or last seen normal time and stroke severity) were consistently reported in a manner allowing enhancing decision-making. Teleconsultation between EMS crews and neurologists is likely to aid with more efficient transportation decisions (i.e., PSC vs. CSC destination) and early activation of stroke intervention teams when indicated and feasible.

Median door-to-needle time (DNT) from all stroke centers within study period was 23 min (IQR 23 min−30 min). Undoubtedly, teleconsultations also contribute to this result.

Accurate decision-making depends on the quality of information provided during the calls. In our study, we found that certain critical pieces of information were provided in the majority of cases (e.g., onset time, severity of neurological deficit, age). However, other important elements, such as anticoagulation status, significant co-morbidities, and premorbid disability were provided inconsistently. Information quality was not associated with the length of teleconsultation.

Communication between paramedics and hospital-based teams is a common practice in medicine. For example, in STEMI cases, EKGs are often transmitted to the hospital and prenotification is provided prior to patient arrival to ensure early mobilization of cardiac catheterization teams, thereby reducing the treatment delays (15). EMS-stroke teleconsultations were highly variable in terms of the quality of provided information. This might be explained by the fact that EMS crews are often faced with certain challenges, including time limitations, environmental factors, and patient factors that can make it difficult to gather and report all relevant information. Similarly, neurologists may have their own “habits” of how they ask for information that may impact if important information is elicited or not. For a future we plan to develop, implement structured checklist-style tool which might be useful to standardize and make these conversations more effective.

The strength of our study is that majority of acute stroke cases is teleconsulted (the least number would be 59% but we conclude from our observations that is much more). On the other hand, the limitation is that we are unable to track disposition endpoint based on the available data and unable to determine how many decisions were “altered” directly due to the teleconsultation itself—largely because this is an established protocol assessed by observational study, so we didn't have a “non-consult” cohort to compare outcomes against.

## Conclusion

In conclusion, teleconsultations represent a feasible tool for stroke triage in prehospital settings. However, inconsistent quality of communicated information presents a potential barrier to optimizing this strategy. Implementation of structured checklist-style communication tool may enhance teleconsultation efficiency by ensuring that all the key information is conveyed and captured. Additional prospective studies examining the utility, cost-effectiveness, and benefit on patients outcomes are needed.

## Data Availability Statement

The raw data supporting the conclusions of this article will be made available by the authors, without undue reservation.

## Ethics Statement

The studies involving human participants were reviewed and approved by University Hospital Ostrava, Czech Republic. Written informed consent for participation was not required for this study in accordance with the national legislation and the institutional requirements.

## Author Contributions

LK, KL, OV, RM, MB, and MH: conceptualization. LK, KL, MC, and DH: formal analysis, data curation, and writing—original draft. OV, RM, and MB: writing—review and editing. OV, MH, MB, and RM: supervision. All authors contributed to the article and approved the submitted version.

## Funding

This work was supported by Ministry of Health, Czech Republic—conceptual development of research organization (FNOs/2018). RM was supported by the COST (European Cooperation in Science and Technology) Association, project No. CA18118, IRENE COST Action – Implementation Research Network in Stroke Care Quality, by the project No. LQ1605 from the National Program of Sustainability II and by the IRIS-TEPUS Project No. LTC20051 from the INTER-EXCELLENCE INTER-COST program of the Ministry of Education, Youth and Sports of the Czech Republic. OV was supported by the INTER-EXCELLENCE grant number LTC20031 – Towards an International Network for Evidence-based Research in Clinical Health Research in the Czech Republic.

## Conflict of Interest

The authors declare that the research was conducted in the absence of any commercial or financial relationships that could be construed as a potential conflict of interest.

## Publisher's Note

All claims expressed in this article are solely those of the authors and do not necessarily represent those of their affiliated organizations, or those of the publisher, the editors and the reviewers. Any product that may be evaluated in this article, or claim that may be made by its manufacturer, is not guaranteed or endorsed by the publisher.
